# Emotional challenges faced by student nurses during clinical placement: A scoping review

**DOI:** 10.4102/hsag.v31i0.3319

**Published:** 2026-03-26

**Authors:** Levon Lubbe, Amanda Dungelo, Kimberly J. Caewood, Ntokozo Mopumulo, Nobuhle Nozakuzaku, Tamar Yazbek, Tshepang Mohlahlo, Nthuseni S. Murudi-Manganye

**Affiliations:** 1 Department of Nursing Sciences, Faculty of Health Sciences, University of Pretoria, Pretoria, South Africa

**Keywords:** emotional challenges, student nurses, clinical placements, resilience, scoping review

## Abstract

**Background:**

Clinical practice is a highly valued learning opportunity to develop the appropriate techniques as student nurses. However, students are experiencing traumatic events during clinical placements without adequate emotional support. This causes emotional challenges that are difficult to manage.

**Aim:**

To explore emotional challenges experienced by student nurses during clinical placements when exposed to traumatic events.

**Method:**

This scoping review followed Arksey and O’Malley’s framework, using the Joanna Briggs Institute (JBI) Manual for Evidence Synthesis to extract and chart sources. Nineteen articles (2014–2024) were analysed using descriptive analysis, Preferred Reporting Items for Systematic reviews and Meta-Analyses extension for Scoping Reviews (PRISMA-ScR) and thematic analysis.

**Results:**

Three themes, namely, psychological resilience, emotional strains and impact on nursing education environment, and coping strategies, emerged from this scoping review. These themes and sub-themes are used to make recommendations.

**Conclusion:**

This scoping review highlights that nursing students face significant emotional challenges, including burnout, insecurity and traumatic experiences, which affect their resilience and well-being. The findings emphasise systemic issues within clinical and academic environments, such as workload pressures, limited support and gaps in preparedness, that exacerbate emotional strain. Positive coping strategies, resilience-building interventions and supportive mentorship were linked to professional growth.

**Contribution:**

The findings from this scoping review might assist in addressing these challenges through curriculum reform, intentional resilience and coping skills training and structured emotional support systems.

## Introduction

Several studies highlight that traumatic event often include witnessing patient deaths, managing critically ill patients, encountering aggressive or violent behaviour from patients or relatives, and dealing with medical errors or near misses (Molefe, 2024; Bacallado-Rodríguez et al., 2025; Cant et al., 2021). Research indicates that such experiences are not rare. A study conducted by Labrague reports that a significant proportion of nursing students, up to 60% – 70% encounter at least one emotionally distressing or traumatic incident during their clinical training (Labrague 2024).

There is a lack of support from experienced professional nurses and clinical facilitators regarding traumatic response management (Hood 2020). This lack of support forces students to deal with the emotional challenges alone, leading to decreased personal and professional growth (Hood 2020). The absence of support causes short-term effects like feelings of panic, anxiety, incompetence, moral distress, compassion fatigue and burnout (Hood 2020), which hinder academic performance and make students reluctant to engage in patient care (Karabey 2023). The lack of emotional validation results in degradation of students’ mental health and can thus reduce their efficacy, leading to reduced quality care and patient satisfaction (Kalyani et al. 2019). Student nurses reported that they experience insecurity during clinical placements, which affects participation and eagerness to experience new opportunities (Ching et al. 2020). Students are hesitant to care for patients who present with traumatic injuries due to the affected well-being after trauma (McCloughen et al. 2020). Gathered reports from student nurses showed continual results of inexperience in dealing with events and the subsequent emotional aftermath (Mbakaya et al. 2020). Along with these short-term effects, it is evident that student nurses are at risk of developing long-term effects.

The frequency of traumatic events, combined with a lack of support, increases the use of ineffective coping mechanisms like desensitisation and dissociation. Research has verified that post-traumatic stress disorder (PTSD) is linked to suicidal ideation, potentially leading to suicide attempts (Kratovic, Smith & Vujanovic 2021).

Student nurses who lack adequate emotional support after traumatic experiences are particularly vulnerable to developing PTSD. Screening based on the DSM-IV criteria has shown positive PTSD rates among student nurses ranging from 15% to 53.3% (Kratovic et al. 2021).

Vulnerable student nurses develop PTSD from traumatic events without emotional support. Student nurses have positive evaluations for PTSD ranging from 15% to 53.3% during screenings according to the DSM-4 model (Kratovic et al. 2021). In the Netherlands, research identified negative clinical experiences and a lack of emotional support as key reasons why student nurses leave the profession (Bakker et al. 2019). In the United States, studies have investigated how exposure to traumatic events can cause psychological trauma, proposing interventions like immediate debriefing and continuous follow-up to mitigate these emotional challenges (Bakker et al. 2019). A similar study in Australia emphasised debriefing as an essential strategy for managing emotions after traumatic experiences (McCloughen et al. 2020).

In a study conducted in Hong Kong, researchers investigated the stressors and coping mechanisms of student nurses, finding that ineffective emotional management and inadequate support from superiors undermined students (Ching et al. 2020). In South Africa, overcrowded hospitals and a shortage of registered nurses further complicate the provision of emotional support to student nurses (Manana et al. 2023). The shortage of staff and the excessive workload affect student nurses’ experiences as newly working staff members and decrease the quality of care (Kovacs & Lagarde 2022).

### Objective of the study

To explore the emotional challenges experienced by student nurses during clinical placements.

### Research question

*How do emotional challenges impact the mental well-being and professional development of student nurses?*

## Methods

This study employed a scoping review methodology to systematically explore and map the existing literature on emotional challenges faced by nursing students in clinical environments. Scoping reviews are designed to provide a broad overview of a topic, identify key concepts and highlight gaps in current research (Pollock et al. 2021). The review was guided by the enhanced framework developed by Arksey and O’Malley, as refined by Levac, Colquhoun and O’Brien (2010), and aligned with the updated methodological guidance from the Joanna Briggs Institute (Peters et al. 2020). This structured approach involved six key stages: (1) framing the review question, (2) establishing eligibility criteria, (3) extracting and collecting data, (4) assessing study quality, (5) summarising the evidence and (6) interpreting the findings (Maggio et al. 2021).

### Framing questions for the review

The review question served as the foundation for the conceptual framework and guided the selection and synthesis of relevant studies. The central question was: How do emotional challenges impact the mental well-being and professional development of student nurses? This question was developed using the revised JBI critical appraisal tool to ensure alignment with the study’s objectives and scope. It enabled a broad yet structured exploration of the literature, allowing for the identification of recurring patterns and meaningful interpretations (Newman & Gough 2020; Campbell 2020). The review adopted a synthesised logic approach to investigate the underlying meaning and implications of emotional challenges within nursing education (Newman & Gough 2020).

### Establishing eligibility criteria

A selection criterion was used during the initial screening to identify resources with relevant information. The eligibility criteria guided the search strategy and determined which studies were suitable for inclusion. Studies were included if they focused on student nurses in clinical environments and were published between 2019 and 2024. Reviews that analysed the impact of traumatic experiences on student nurses were considered, along with studies showing both short- and long-term effects on patient care. Excluded materials were those lacking evidence about affected students or focusing on postgraduate nurses. Articles that only provided abstracts, required payment or were duplicates were also excluded. Full thesis studies and non-peer-reviewed articles were not considered. Only English-language articles were included to ensure consistency in analysis.

### Extraction and collecting data

A multi-dimensional data extraction process was conducted using the Covidence tool, managed by the University of Pretoria, with the scoping review registered on 31 March 2025. Following best practices outlined by Martinez et al. (2023), the review involved multiple reviewers analysing both adjusted and non-adjusted data. All reviewers accessed the full-text stage and used a standardised data extraction template to record key study details such as author, publication year, country, study design, population characteristics, interventions, outcomes and findings. This template was pilot tested and refined to ensure consistency across reviewers. A parallel quality assessment template was developed to evaluate the risk of bias, using Covidence’s customisable checklists. Each study was independently assessed by two reviewers, with discrepancies resolved within the platform by the supervisor in collaboration with the student reviewers.

The search strategy was developed using key terms derived from the review topic and refined with Boolean operators such as AND, OR and NOT. Databases used included PubMed, ScienceDirect, Google Scholar and Web of Science, all approved by a research specialist from the University of Pretoria. The search terms combined concepts like ‘student nurses’ OR ‘nursing students’ OR ‘nurse trainees’ with ‘challenges’ OR ‘stressors’ OR ‘barriers’ and ‘clinical placement’ OR ‘clinical rotation’ OR ‘practicum.’ Optional refinements included emotional support and the study period to narrow results. Covidence facilitated the initial screening of titles and abstracts, which were then reviewed by seven nursing students. The supervisor served as the final reviewer, resolving conflicts and ensuring quality control throughout the review process.

### Evidence screening and selection

Following the search, every citation found was gathered and added to the supervisors’ registered Covidence account. The chosen sources for the scoping review were then managed and arranged, and duplicates were removed by importing the references into the Covidence tool. The researchers assessed the abstracts and titles, comparing them with the researchers’ inclusion standards. All possibly relevant sources were retrieved in full, and their citation data were included. The researcher thoroughly reviewed and assessed all the selected sources to ensure they met the criteria for inclusion. Full-text sources of evidence that did not meet the inclusion criteria were excluded, and the scoping review noted and recorded the rationale for these decisions.

### Charting the data

A logical and descriptive summary of the outcomes is provided by data extraction or data charting, which also shows how well the results match the goals of the scoping review. The literature about the emotional challenges experienced by nursing student during clinical placement was the main topic of the data that were extracted. A data-extraction tool that was modified from the JBI Manual for Evidence Synthesis served as guidance for the data extraction or charting procedure (Barker et al. 2023). Table 1 illustrates how the tool was used to extract information from the publications about the scoping review, including authors, publication year, source details, methodology and important conclusions.

**TABLE 1:** Characteristics of the studies included in the scoping review.

Author	Year	Study title	Country	Population	Setting	Study design
Shirley Siu Yin Ching and Kin Cheung	2020	Factors affecting resilience of nursing, optometry, radiography, and medical laboratory science students	Hong Kong	Nursing, Optometry, Radiography, and Medical Laboratory Science students (*n* = 1320)	The Hong Kong Polytechnic University	Cross-sectional quantitative survey study
Shirley Siu Yin Ching, Kin Cheung, Desley Hegney, Clare S Rees	2020	Stressors and coping of nursing students in clinical placement: A qualitative study contextualizing their resilience and burnout	Hong Kong	4th-year baccalaureate nursing students	The Hong Kong Polytechnic University – Clinical placements	Qualitative study
Chun-Chih Lin and Li-Chin Chen	2023	An exploration of psychological resilience among undergraduate nursing students undertaking an adult nursing virtual practicum during the coronavirus pandemic	Taiwan	Undergraduate nursing students	Virtual practicum	Qualitative study
Shirley D. Martin et al.	2022	Health-related behaviors, self-rated health, and predictors of stress and well-being in nursing students	United States (North Texas)	Baccalaureate Nursing students	Texas Christian University, University of Texas Arlington, Texas Women’s University	Multi-site survey-based study
Winnie Lai-Sheung Cheng et al.	2024	Examining the effects of moral distress, compassion fatigue and burnout on intention to leave among nursing students	Hong Kong	480 Nursing students	Nine educational institutions in Hong Kong	Cross-sectional correlational study
Tuba Karabey	2023	Compassion fatigue and psychological resilience levels of nursing final students	Turkey	250 final-year nursing students	Online survey (Türkiye)	Descriptive, cross-sectional, relational study
Yuanyuan Zhang et al.	2023	Prevalence of compassion fatigue and its association with professional identity in junior college nursing interns	China	2256 nursing interns	Junior nursing colleges (online survey)	Descriptive, cross-sectional study
Labrague et al.	2025	Psychological resilience as a mediator between nurse faculty support and student nurses’ clinical adjustment	Philippines	506 student nurses	Three nursing schools	Correlational quantitative study
Lebogang Molefe	2024	R425 first year student nurses’ experience of encounters with death of a patient during clinical placement	South Africa	15 first-year student nurses	Sefako Makgatho Health Sciences University	Qualitative exploratory descriptive and contextual research design
Ellen J.M. Bakker	2019	Late dropout from nursing education: An interview study of nursing students’ experiences and reasons	Netherlands	3rd year student nurses	Universities (interviews, housing, Skype)	Exploratory qualitative design
Asha K. Nabirye et al.	2025	Emotional and psychological experiences of nursing students caring for dying patients: An explorative study	Uganda	15 nursing students	Mulago National Referral Hospital	Qualitative explorative study
Kobra Ghorbanzadeh et al.	2023	Explaining the clinical education stressors in nursing students: A qualitative study	Iran	16 nursing students	Khalkhal University of Medical Sciences	Qualitative content analysis
Emre Ciydem	2024	The relationship between difficulties in emotion regulation and solution-focused thinking in nursing students	Türkiye	416 nursing students	Nursing faculties in Türkiye	Cross-sectional, descriptive and correlational study
Pimwalunn Aryuwat	2024	Experiences of nursing students regarding challenges and support for resilience during clinical education	Thailand	28 nursing students	Clinical placements	Qualitative study
Andrea McCloghen	2020	Nursing students’ socialization to emotion management during early clinical placement experiences	Australia	First-year nursing students	Clinical placements	Qualitative study
Beauty M. Zulu et al.	2021	Experiences of nursing students regarding clinical placement and support in primary healthcare clinics: Strengthening resilience	South Africa	25 fourth-year nursing students	Primary healthcare clinics	Qualitative, exploratory, descriptive and contextual research design

Note: Please see full reference list of this article: Lubbe, L., Dungelo, A., Caewood, K.J., Mopumulo, N., Nozakuzaku, N., Yazbek, T. et al., 2026, ‘Emotional challenges faced by student nurses during clinical placement: A scoping review’, *Health SA Gesondheid* 31(0), a3319. https://doi.org/10.4102/hsag.v31i0.3319 for more information.

### Assessing study quality

The data analysis in this scoping review followed the three-step process outlined in the framework by Arksey and O’Malley (2005). The first step involved developing an analytical framework using the PRISMA-ScR flow diagram to provide a comprehensive overview of the literature related to emotional challenges affecting nursing students’ learning. This visual tool helped map the selection process and ensured transparency in study inclusion. The second step applied descriptive statistical methods, with data presented in tabular format to illustrate key study characteristics such as author, publication year, study title and country of origin (Table 1). This helped categorise the types of research and identify trends across the selected studies. The final step employed thematic analysis to explore recurring patterns and concepts related to emotional challenges during clinical placements. Themes and sub-themes were identified and examined in depth to provide insight into the emotional experiences of nursing students.

A significant portion of the studies employed qualitative designs, with 13 studies using approaches such as exploratory, descriptive, contextual or content analysis methods. These qualitative studies aimed to understand nursing students’ lived experiences, emotional responses and coping mechanisms during clinical placements. In contrast, cross-sectional quantitative designs were employed in five studies, including survey-based and correlational approaches, which aimed to measure variables such as resilience, stress and compassion fatigue across large student populations. Additionally, descriptive and relational studies were employed in two instances to explore the associations between psychological factors and student outcomes. One study employed a multi-site survey-based design, and another utilised a correlational quantitative approach to examine the relationships between faculty support and student adjustment.

### Interpreting the findings

**FIGURE 1:**
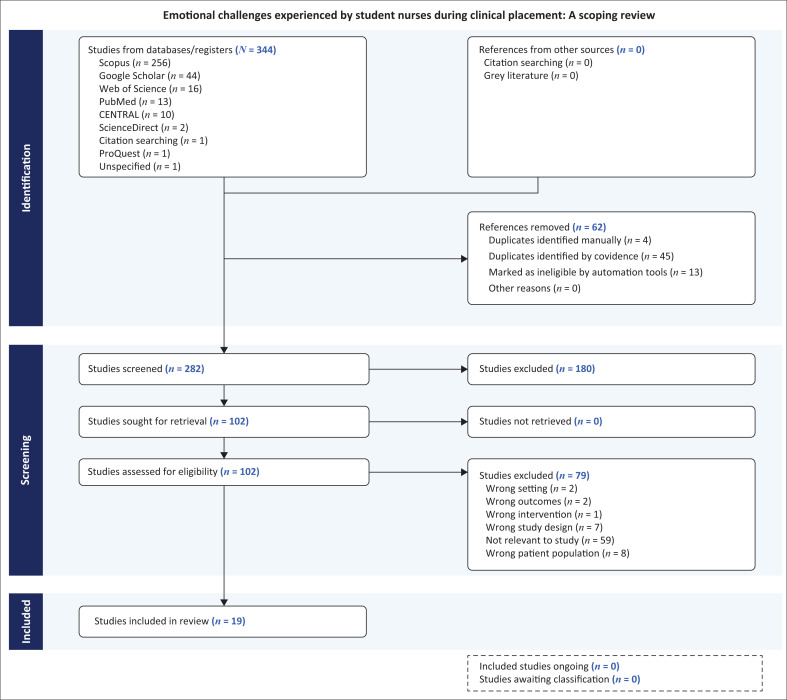
Preferred Reporting Items for Systematic Reviews and Meta-Analyses 2020 flow diagram showing the study selection process, generated using Covidence systematic review software.

A total of 344 studies were initially identified across eight databases, with 62 removed because of duplication and ineligibility. After screening 282 studies, 180 were excluded based on the predefined exclusion criteria. Of the remaining 102 full-text articles, 83 were excluded, leaving 19 studies that met the inclusion criteria. These studies were interpreted, summarised and reported using a Preferred Reporting Items for Systematic Reviews and Meta-Analyses (PRISMA) flow diagram (Figure 1), developed with the Covidence tool and aligned with Open Science framework standards. The final selection included a mix of study designs: 13 qualitative studies, five cross-sectional quantitative studies and one correlational quantitative study. The qualitative studies explored lived experiences, emotional responses and coping strategies, while the quantitative studies measured resilience, stress and psychological outcomes.

The included studies examined a range of emotional challenges and coping strategies among student nurses during clinical placements. Several studies focused on stressors and resilience, with the largest contribution coming from Hong Kong (three studies). Other research explored end-of-life care and inadequate preparation, as seen in studies from South Africa and Iran. Investigations from the United States examined health-related behaviours and coping mechanisms among baccalaureate students, while studies from Taiwan, Turkey, China and the Philippines addressed compassion fatigue, resilience and curriculum alignment. Additional studies from the Netherlands, Uganda, Thailand and Australia highlighted emotional exposure and the role of support systems.

Thematic analysis revealed three overarching themes and 17 sub-themes, identified through repeated patterns and structural meanings in the data. These themes reflect the global nature of emotional challenges in nursing education, shaped by cultural, institutional and healthcare contexts. Table 2 outlines these themes, emphasising the importance of resilience, support and preparedness in nursing education.

**TABLE 2:** Themes and sub-themes.

Theme 1: Psychological resilience and emotional strain	Theme 2: Impact of nursing education, environment and educators	Theme 3: Coping strategies to manage emotional challenges
Emotional fatigue, compassion fatigue and burnout	Academic stress and performance pressure	Adaptive approaches
Uncertainty, insecurity and self-doubt	Environmental challenges	Maladaptive approaches
Emotional exposure	Emotional support from preceptors, mentors, peers and faculty	Role of health-related behaviours
Impact of workload and staff shortages	Gaps in preparation	Social support
Interpersonal challenges	Curriculum design and alignment with practice	Adjustment through repeated exposure
High vs. low resilience coping mechanisms	Expectations vs reality in clinical placement	-

### Ethical considerations

An application for full ethical approval was made to the University of Pretoria Faculty of Health Sciences ethics committee and ethics consent was received on 20 November 2024. The ethics approval number is 697/2024.

## Summarising the evidence

The 19 articles included in this scoping review were published between 2019 and 2025. Table 1 presents a detailed distribution of publication years, illustrating a consistent growth in research interest over the period. One article was published in 2019, followed by three in 2020, and two in 2021. The year 2022 saw another three publications, maintaining the upward trend. In 2023, three more studies were added, and the most recent year, 2025, contributed four articles, indicating growing attention to the topic. These studies originated from 13 different countries, with Hong Kong contributing the highest number (three studies), followed by South Africa with two. Other countries such as Iran, Taiwan, Turkey, China, the Philippines, the Netherlands, Uganda, Thailand and Australia each contributed one study. This geographical spread highlights the global relevance of emotional and psychological challenges faced by nursing students, though regional differences in focus and methodology were evident.

### Theme 1: Psychological resilience and emotional strain

Nursing students frequently reported emotional fatigue, compassion fatigue and burnout, particularly in high-pressure clinical environments. In Hong Kong, moral distress and burnout were significantly linked to students’ intention to leave nursing programmes (Cheng, Tang & Siu 2024), with burnout being the most prevalent, followed by compassion fatigue and moral distress. Similarly, compassion fatigue was associated with lower resilience among final-year nursing students in Turkey (Karabey 2023). Feelings of insecurity and self-doubt were common when students entered unfamiliar wards or faced complex patient conditions, as seen in Ireland’s neonatal units, where preceptor support helped reduce stress (Mulligan & Frawley 2022). Iranian nursing students reported experiencing identity confusion related to their transition into the professional nursing role and used diverse coping strategies to manage challenges in clinical settings (Kalyani et al. 2019).

Iranian students experienced identity confusion and diverse mixed coping strategies in clinical settings (Kalyani et al. 2019), while Thai students emphasised the importance of supervisor support in building confidence (Aryuwat et al. 2024). Emotional exposure to patient death and suffering triggered intense reactions such as fear and helplessness, especially among South African first-year students who lacked adequate preparation (Molefe 2024). Ugandan students relied on spirituality and peer support to manage these experiences (Nabirye et al. 2025), whereas Australian students were left to self-reflect because of insufficient guidance (McCloughen et al. 2020). These findings highlight the need for structured emotional support and debriefing in nursing education.

Resilience is commonly understood as the ability to adapt positively and recover from adversity, stress or trauma by demonstrating mental, emotional and behavioural flexibility and adjustment to challenging circumstances (American Psychological Association [APA] 2023; NursesLab 2023). Studies reported that workload and staffing shortages were major stressors, often requiring students to balance patient care with academic demands, which led to exhaustion and frustration (Molazem, Joghataei & Nikbakht-Nasrabad 2019; Mulligan & Frawley 2022). Heavy clinical hours contributed to compassion fatigue and doubts about long-term commitment to the profession (Karabey 2023; Martin et al. 2022). Interpersonal relationships with staff and preceptors also played a role, with negative interactions causing feelings of exclusion and insecurity (McCloughen et al. 2020; Molazem et al. 2019), while supportive mentorship fostered confidence and growth (Labrague et al. 2025; Zulu, Du Plessis & Koen 2021). Students with higher resilience demonstrated adaptive strategies such as mindfulness and emotional regulation (Ching et al. 2020; Lin & Chen 2023), whereas those with lower resilience tended to rely on avoidance and external validation (Ching et al. 2020; Watson et al. 2025). Overall, the studies indicate that resilience, along with stressors and support systems, plays a significant role in shaping emotional and clinical experiences during placements.

Workload and staffing shortages were major stressors affecting students’ resilience during clinical placements. Students often had to balance patient care with academic demands, leading to exhaustion and frustration (Molazem et al. 2019; Mulligan & Frawley 2022). Heavy clinical hours contributed to compassion fatigue and doubts about long-term commitment to the profession (Karabey 2023; Martin et al. 2022). Interpersonal relationships with staff and preceptors also shaped resilience, with negative interactions causing feelings of exclusion and insecurity (McCloughen et al. 2020; Molazem et al. 2019), while supportive mentorship fostered confidence and growth (Labrague et al. 2025; Zulu et al. 2021). Students with high resilience used adaptive strategies like mindfulness and emotional regulation (Ching et al. 2020; Lin & Chen 2023), whereas those with low resilience relied on avoidance and external validation (Ching et al. 2020; Watson et al. 2025). These differences illustrate how resilience influences coping strategies, with high-resilience students using adaptive approaches and low-resilience students relying on avoidance and external validation.

Resilience mediates stress and coping, influencing students’ ability to manage challenges effectively. Overall, Theme 1 reveals the complex interplay between emotional strain, resilience and support systems in shaping nursing students’ clinical experiences.

### Theme 2: Impact of nursing education, environment and educators

Nursing students face significant academic stress and performance pressure, as they are expected to demonstrate theoretical competence while simultaneously applying it in clinical practice without prior exposure (Ghorbanzadeh et al. 2023). This dual expectation often leads to anxiety and self-doubt, especially when students are required to manage unfamiliar patient conditions independently. Environmental challenges further compound these pressures, with students frequently placed in unfamiliar clinical settings without adequate orientation or debriefing (Lin & Chen 2023). The lack of guidance in patient-care approaches contributes to feelings of uncertainty and frustration (Lin & Chen 2023). Clinical placement factors such as fear of medical errors, lack of skills and emergency situations overwhelm students (Ching et al. 2020; Lin & Chen 2023). Emotional support from mentors, preceptors and faculty is often inconsistent, leaving students feeling neglected during critical learning moments (Mulligan & Frawley 2022). Without encouragement, students struggle to connect theory with practice, leading to burnout and early withdrawal from nursing programmes (Bakker et al. 2019). These findings highlight the importance of structured mentorship and emotional support in clinical education.

Gaps in student preparation were evident, particularly in areas such as emotional intelligence, emotion regulation and end-of-life care (Molefe 2024; South African Nursing Act 2019). Students reported receiving inadequate education on death-related topics, leaving them unprepared for emotionally intense clinical experiences (Nabirye et al. 2025). As a result, they were forced to bridge the gap between theory and practice independently, often without sufficient guidance (Ching et al. 2020; Najafi Kalyani et al. 2019). Curriculum design and alignment with clinical practice emerged as a critical factor in promoting resilience and reducing stress. Studies emphasised the need for curriculum adjustments to better prepare students for real-world challenges (Ghorbanzadeh et al. 2023; Karabey 2023; Williams-Jones 2023). Students also reported a mismatch between expectations and the realities of clinical placements, which negatively impacted their perceptions and learning outcomes (Ching et al. 2020). This challenge often stems from curricula that place greater emphasis on theoretical knowledge while providing limited opportunities for hands-on practice and insufficient integration of theory with real-world clinical experiences. As a result, students struggle to apply classroom learning effectively in unpredictable clinical settings.

The unpredictable nature of clinical environments often undermines the effectiveness of theoretical education (Ghorbanzadeh et al. 2023).

### Theme 3: Coping strategies to manage emotional challenges

Nursing students employed a variety of adaptive coping strategies to manage the emotional demands of clinical placements. Techniques such as mindfulness, physical exercise, self-reflection and spirituality were commonly used to maintain emotional balance and resilience (Ching et al. 2020; Martin et al. 2022; Nabirye et al. 2025; Watson et al. 2025). Positive reframing, acceptance and humour were found to enhance students’ ability to adapt to demanding environments (Ching et al. 2020). Spiritual practices and peer support offered emotional comfort, while self-care routines helped strengthen psychological well-being (Nabirye et al. 2025; Watson et al. 2025). These findings support the integration of mindfulness training, reflective opportunities and wellness activities into nursing curricula to reduce burnout and promote long-term resilience. In contrast, maladaptive coping strategies such as avoidance, emotional disengagement and denial were also reported. These behaviours were linked to increased psychological distress and reduced resilience (Ching et al. 2020; Molazem et al. 2019; Nabirye et al. 2025). Students who relied on avoidance were more vulnerable to compassion fatigue and burnout, highlighting the need for early intervention and mentorship to promote healthier coping mechanisms.

Health-related behaviours such as sleep, diet and exercise were consistently identified as key contributors to emotional resilience. Students who maintained positive lifestyle habits were better equipped to handle clinical stress, while poor habits increased vulnerability to anxiety and fatigue (Martin et al. 2022; Watson et al. 2025). Sleep deprivation and disrupted routines were perceived as barriers to coping, often leading to emotional instability and reduced competence (Watson et al. 2025).

Social support from faculty, mentors and peers played a vital role in strengthening resilience and promoting emotional well-being (Aryuwat et al. 2024; Labrague et al. 2025; Zulu et al. 2021). Supportive supervisors fostered collaboration and empathy, while preceptors ensured safety and guidance during emotionally taxing tasks (Mulligan & Frawley 2022). Repeated exposure to clinical environments also contributed to emotional adjustment, with students developing confidence and problem-solving skills over time (Ching et al. 2020; Ghorbanzadeh et al. 2023; Lin & Chen 2023; Molazem et al. 2019; Zulu et al. 2021). Junior students were more likely to struggle, while senior students demonstrated greater emotional stability.

## Discussion

The emotional and psychological challenges faced by nursing students during clinical placements are multifaceted. Emotional fatigue, burnout and compassion fatigue were prevalent, often exacerbated by workload and clinical pressure (Cheng et al. 2024; Karabey 2023; Fadana & Vember 2021). These stressors were compounded by feelings of insecurity and self-doubt, particularly in unfamiliar clinical environments (Aryuwat et al. 2024; Mulligan & Frawley 2022). Exposure to patient death and suffering further intensified emotional strain, revealing gaps in emotional preparedness and support systems (Molefe 2024; Nabirye et al. 2025). Evidence suggests that resilience, defined as the ability to adapt positively and recover from adversity, plays a critical role in coping, with high-resilience individuals employing adaptive strategies such as mindfulness and emotional regulation, while low-resilience students are more prone to disengagement and burnout (Ching et al. 2020; Watson et al. 2025). These findings indicate that resilience is not merely a personal trait but a skill that can be strengthened through structured interventions.

Academic stress and performance pressure also emerged as significant contributors to emotional strain, as students are expected to apply theoretical knowledge in high-stakes clinical settings without adequate preparation (Ghorbanzadeh et al. 2023). Environmental challenges, such as a lack of orientation and guidance, further undermine students’ confidence and increase stress (Ching et al. 2020; Lin & Chen 2023). The absence of emotional support from mentors and faculty leads to feelings of isolation and disconnection from the profession, contributing to early withdrawal (Bakker et al. 2019; Mulligan & Frawley 2022). Gaps in curriculum design, particularly in areas like emotional intelligence and end-of-life care, leave students ill-equipped to manage clinical realities (Molefe 2024; Nabirye et al. 2025). The misalignment between theoretical instruction and practical application underscores the need for curriculum reform to better prepare students for clinical demands (Karabey 2023; Williams-Jones 2023). Curriculum reform can be driven by embedding emotional intelligence and end-of-life care modules into core courses, incorporating simulation-based learning for emotionally challenging scenarios and ensuring stronger alignment between theory and practice through extended clinical rotations and structured debriefing sessions. Collaboration between academic institutions and clinical partners is essential to implement these changes and provide consistent emotional support systems.

Coping strategies identified in this review offer practical interventions to mitigate emotional strain and enhance resilience. Positive strategies such as mindfulness, physical activity and peer support were associated with reduced stress and improved well-being (Martin et al. 2022; Nabirye et al. 2025; Johnson et al. 2023). In contrast, maladaptive approaches like avoidance and emotional disengagement were linked to increased psychological distress and burnout (Ching et al. 2020; Molazem et al. 2019). Health-related behaviours, including sleep, diet and exercise, significantly influenced students’ ability to cope, with poor habits exacerbating emotional instability (Watson et al. 2025). Social support from faculty, mentors and peers emerged as a critical buffer against emotional strain, fostering resilience and professional growth (Labrague et al. 2025; Zulu et al. 2021). Repeated exposure to clinical environments also contributed to emotional adjustment, with senior students demonstrating greater stability and confidence (Ghorbanzadeh et al. 2023; Lin & Chen 2023). These findings reinforce the importance of integrating wellness education, structured mentorship and progressive clinical exposure into nursing programmes to support student well-being and retention.

### Recommendations

To enhance nursing students’ experiences, recommendations should directly address the emotional, educational and support gaps identified in this review. Firstly, curricula should be revised to better integrate theory and practice through simulation-based learning, extended clinical rotations and structured debriefing sessions. These changes will reduce educational gaps and improve students’ preparedness for emotionally demanding situations. Secondly, targeted education on coping mechanisms such as mindfulness, emotional regulation and peer support should be embedded in nursing programmes. Faculty and mentor-led support systems, including regular supervision and reflective practice, are essential for fostering resilience and reducing stress. Thirdly, resilience-building strategies should be incorporated into training, as resilience is linked to improved coping and reduced burnout. Education on trauma and end-of-life care must also be prioritised to prepare students for challenging clinical realities. Fourthly, wellness initiatives focusing on sleep, nutrition and physical activity should complement academic training to promote holistic well-being and professional development.

### Limitations

This review was limited by its reliance on six databases, which may have introduced selection bias and restricted the scope of the findings. Consequently, the ability to generalise the results regarding emotional challenges faced by nursing students during clinical placements may be constrained. Additionally, the exclusion of non-English articles and journals may have narrowed the breadth of available evidence, potentially omitting valuable insights from non-English-speaking regions. The screening process, while rigorous, may have inadvertently excluded relevant studies because of language barriers. Another limitation is the absence of a deep critical appraisal of the included studies, which may have affected the depth and robustness of the conclusions drawn. The descriptive and thematic analyses provided a broad overview but lacked a detailed evaluation of methodological quality. These limitations should be considered when interpreting the findings and applying them to broader educational or clinical contexts.

## Conclusion

This scoping review aimed to explore the emotional challenges encountered by nursing students during clinical placements. This scoping review demonstrates that student nurses experience emotional challenges during clinical placements, including burnout, compassion fatigue and insecurity. These challenges affect their mental well-being, academic performance and professional development and cause attrition. The review highlights systemic factors like heavy workloads, staff shortages, inadequate preparation and insufficient emotional support. Despite these difficulties, resilience tools and supportive mentorship were consistently associated with adaptation and growth. The findings underscore the importance of integrating support systems and resilience training into nursing curricula, alongside mentorship and debriefing opportunities. Addressing these issues promotes well-being and professional preparedness and strengthens the sustainability of the nursing workforce. Future research within the South African context is needed to explore localised stressors and to develop evidence-based interventions that respond to the realities of nursing education and healthcare environments.
